# A Scoping Review of Trauma-Informed Pediatric Interventions in Response to Natural and Biologic Disasters

**DOI:** 10.3390/children10061017

**Published:** 2023-06-05

**Authors:** Kimberly Burkhart, Neel Agarwal, Sehyun Kim, Mandy Neudecker, Carolyn E. Ievers-Landis

**Affiliations:** 1 Rainbow Babies and Children’s Hospital, Case Western Reserve University School of Medicine, 10524 Euclid Ave., Cleveland, OH 44106, USA; carolyn.landis@uhhospitals.org; 2 Case Western Reserve University, 10900 Euclid Ave., Cleveland, OH 44106, USA; nxa357@case.edu (N.A.); sxk1502@case.edu (S.K.); 3 Rainbow Babies and Children’s Hospital, 1100 Euclid Ave., Cleveland, OH 44106, USA; mandy.neudecker@uhhospitals.org

**Keywords:** disasters, pandemic, pediatric, psychosocial treatment, review

## Abstract

A scoping review was performed of trauma-informed psychological interventions to treat anxiety, depression, and posttraumatic stress symptoms in youth in response to natural/biologic disasters. The specific aims were to identify psychosocial interventions used in response to natural/biologic disasters, report the interventions’ effectiveness, describe limitations, and provide treatment recommendations and future directions. Of the 45 studies extracted, 28 were on natural disasters and 17 on biologic disasters with the majority related to the COVID-19 pandemic. The most commonly implemented interventions were Cognitive Behavioral Therapy (CBT), Trauma-Focused Cognitive Behavioral Therapy (TF-CBT), and eye movement desensitization and reprocessing (EMDR). The UCLA Posttraumatic Stress Disorder Reaction Index (UCLA PTSD-RI) and the Strengths and Difficulties Questionnaire (SDQ) were the most frequently used measures. Methodological rigor was varied, with 60% randomized, controlled trials. Overall, there was a significant decrease in posttraumatic stress symptoms, distress, anxiety, and depression regardless of whether the participant received CBT, TF-CBT, or EMDR. Generally, there was not a significant decrease in anxiety and depression with yoga, cognitive fear-reduction, emotion-based drawing, and community health education. Recommendations for future directions include larger-scale studies with group and on-line interventions that include younger children with moderation analyses by gender and race/ethnicity.

## 1. Introduction

Disasters can be categorized as either manmade or natural/biologic with the majority of studies exploring the efficacy of intervention in response to manmade disasters such as terrorism and war. There has been a significant increase, however, in natural disasters (e.g., floods, wildfires, hurricanes, earthquakes, and tsunamis) and biological events (e.g., pandemics) during the 21st century [1]. Millions of children were affected by the COVID-19 pandemic with over 15.5 million testing positive for this infectious disease and over 100,000 US children losing a primary caregiver to the pandemic [2]. Such traumatic exposures can be associated with a higher risk of developing post-traumatic stress disorder (PTSD), depression, anxiety, behavior problems, substance use disorders, suicidal ideation/self-harm, poorer academic and occupational achievement, and adverse physical outcomes [3,4].

Professionals in emergency medicine have identified five phases of the disaster cycle: prevention, mitigation, preparedness, response, and recovery. As it relates to behavioral health, the *prevention phase* consists of identifying threats/risks and sources of community resilience that could serve to minimize the psychological impact of a disaster. The *mitigation phase* involves taking steps to prevent and reduce the cause, impact, and potential consequences of future disasters. In the *preparedness phase*, behavioral health clinicians assist with evaluation and quality improvement to ensure everyday readiness. The *response phase* is focused on restoring optimal psychological well-being and functioning through both short- and long-term behavioral health interventions. And finally, in the *recovery phase,* the goal is to stabilize and restore all behavioral health supports, returning children and their families to their pre-disaster level of functioning, and/or creating a new normal [5].

The intent of this review is to identify interventions that can be used in the response phase of a natural/biologic disaster and to use the information obtained to inform recommendations for preparedness. To date, there has been a lack of specific focus on the efficacy and effectiveness of child disaster mental health interventions in response to such disasters.

Mental health treatments shown to be effective in the treatment of trauma-exposed youth include trauma-focused cognitive behavioral therapy (TF-CBT), trauma and grief component therapy for adolescents (TGCT-A), and eye movement desensitization and reprocessing (EMDR). TF-CBT is an evidence-based manualized treatment that consists of nine cognitive and behavioral components [6]. These components include (1) psychoeducation, (2) parenting skills, (3) relaxation skills, (4) affective modulation skills, (5) cognitive processing skills, (6) trauma narration and processing, (7) in-vivo mastery of trauma reminders, (8) conjoint parent-child sessions, and (9) enhancing future safety. TGCT-A is an evidence-based modular approach to treat trauma, bereavement, and traumatic bereavement. The four modules in this intervention include providing foundational knowledge about trauma and skill building (Module 1), working through traumatic experiences (Module 2), working through grief experiences (Module 3), and looking to the future to identify developmental disruptions and set realistic expectations and plans for the future (Module 4) [7]. EMDR has been established as an effective intervention in the treatment of children/adolescents. EMDR is an eight-phase approach rooted in the adaptive information-processing model with the objective of desensitizing the patient to an unpleasant memory by reliving the trauma without experiencing the sensations of imminent threat [8]. Although all are empirically supported interventions, it is unclear as to the frequency with which they are implemented in response to distress associated with experiencing a natural/biologic disaster. Other possible interventions should be explored due to the potential lack of access to clinicians trained in these approaches and possible barriers to accessing services.

To the authors’ knowledge, this is the first scoping review to focus solely on natural/biologic disasters and to include a specific focus on interventions used in response to the COVID-19 pandemic. It is also the first review to include evaluation of the efficacy of interventions on internalizing symptoms in addition to posttraumatic stress in response to a disaster. Our specific aims of this review are to: (1) identify psychosocial interventions that are used in response to natural/biologic disasters, (2) report on the efficacy and effectiveness of the identified interventions, (3) identify gaps/limitations, (4) provide treatment recommendations based on this review, and (5) identify strategies for enhancing research in this field.

## 2. Materials and Methods

### 2.1. Search Strategy

A scoping review approach was chosen as it was anticipated that there would be a diverse body of literature pertaining to the broad topic of natural disasters. A search strategy was generated to capture the concepts of psychological interventions used to treat children in response to natural disasters. The search included database-controlled vocabulary and text word searching with Boolean operators. Unpublished literature, dissertations, conference proceedings, and registered trials were included in the results from the databases. A hand search of the most relevant articles reviewed in the full text was completed. Terms for natural disasters were weather or COVID-19 pandemic related. Pediatric terms were used to limit results to youth and transitional-aged youth. The search was limited to English. Studies published between 2000 and 2023 were included.

Titles and abstracts of articles identified via the search terms were uploaded to the online platform Covidence. See Table 1 for abstracts imported to Covidence for review. Once uploaded, two reviewers followed the built-in protocols to screen identified articles in the following manner: (1) Screen title and abstract, (2) Full text review screen, and 3) Article extraction. A third rater on the research team addressed disagreements as to the inclusion/exclusion criteria.

### 2.2. Study Selection

**Figure 1:**
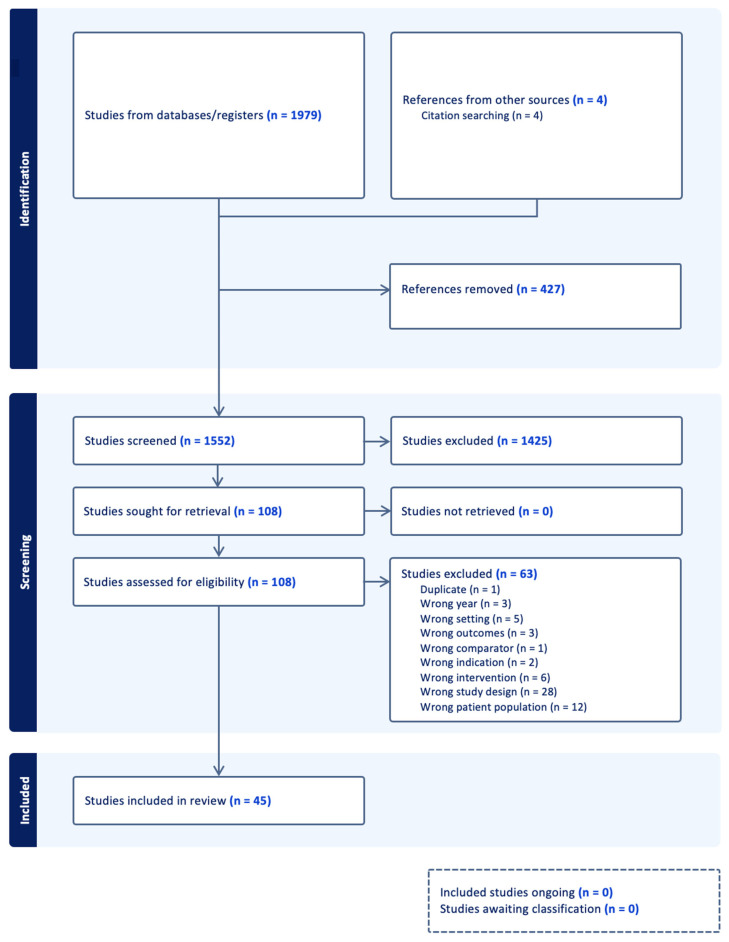
PRISMA flowchart of paper selection.

The study selection PRISMA flowchart is presented in Figure 1. The combined searches initially yielded 1979 articles. Duplicates were removed (N = 427). Whereas a total of 1552 studies were screened, 45 studies met criteria to be included in the review.

## 3. Results

### 3.1. Overview of Extracted Articles

Of the 45 studies extracted, 28 were published on natural disasters (earthquake, n = 11; flood, n = 2; hurricane, n = 8; typhoon, n = 1; tornado, n = 2; tsunami, n = 3; hurricane/earthquake, n = 1), and 17 were published on biologic disasters (AIDS, n = 2; COVID-19, n = 15). The majority were therefore conducted on the COVID-19 pandemic. See Table 2.

### 3.2. Demographics of Participants

The age of children and adolescents included in these studies ranged from 5 to 19 years, including three studies with emerging adults who were as old as 20 to 24 [27,29]. The majority of the studies included only adolescents (33/45, 73.3%). This is interesting as the recommended age for the therapies tested includes younger children. For example, TF-CBT has been rated as “probably efficacious” for preschool-aged children as young as 3 years old who have at least some memory of their trauma [53]. In addition, self-report measures of trauma typically start between ages 6–8 years. This may partially explain why very young children were not included. The age range varied between studies, with some including children within a 2-year age range and others including a large age range [14,21,27,38]. For studies that reported sex, females were included at slightly higher percentages than males (overall, 5284/8383 = 63%). This seems reasonable considering that females tend to have a higher risk of PTSD after exposure to a disaster, such as found in a systematic review of children and adolescents after earthquakes and floods [54].

In the U.S., the race/ethnicity of the participants was reported as African American/Black, White/non-Hispanic White/Caucasian, Latino/Latinx/Hispanic, with fewer studies reporting percentages of Asian, Native American/American Indian/Alaska Native, Native Hawaiian/Other Pacific Islander, or biracial/multiracial/other racial/ethnic backgrounds. Three U.S. studies did not include race/ethnicity data, with one in Hawaii, the other in Puerto Rico, and the third in New Orleans. Reporting standards have recently been proposed for ethnicity/race for journal articles, which include not collapsing across categories and avoiding the assumption that whiteness is the norm [55]. In terms of geographic region, the studies took place in multiple countries in 6 of the 7 continents, including Africa (South Africa), Asia (China, Iran, Japan), Europe (Italy, Spain), North America (Canada, Mexico, United States), Australia/Oceania (Australia, New Zealand), and South America (Argentina).

### 3.3. Study Design Features

Sample sizes varied greatly, with 25/45 (55.5%) having fewer than 100 participants. One of these small n studies was of 31 children affected by the tsunami in Sri Lanka [11]. There were two large-scale research studies of more than a thousand adolescents. One was a group after-school intervention of 1030 adolescents (545 intervention/550 control) following Hurricane Hugo in which the control group did not receive an intervention [10]. The other was an on-line single-session randomized controlled trial of more than 2000 adolescents during the COVID-19 pandemic [15].

The studies varied in their methodological rigor. Slightly more than half (26/45; 58%) were randomized controlled trials. Some of these trials compared two experimental groups (n = 10, 38%), others including control groups received either a standard intervention (n = 8, 31%) or were a no-treatment control (n = 8; 31%); somewhat surprisingly, only four studies reported using a wait-list control group [9,50].

### 3.4. Description of the Constructs and Measures

Constructs most commonly assessed included screening for PTSD symptoms, reactions to trauma, depressive symptoms, anxiety, general psychological adjustment, and self-efficacy. The most commonly assessed construct was PTSD symptoms, with 20/45 studies (44%) including such measures. Constructs less often explored in these studies were subjective units of distress, social support, hopelessness, cognitive functioning, health-related quality of life, psychological flexibility, experiential avoidance, post-traumatic growth, negative life events, and overall level of fearfulness.

Multiple measures were used in at least three studies. One of the most often used measures, albeit not focused specifically on trauma symptoms, was the Strengths and Difficulties Questionnaire (SDQ) [56]. The SDQ is a 25-item questionnaire that assesses emotional symptoms, conduct problems, hyperactivity/inattention, peer relationship problems, and prosocial behavior. The SDQ has been normed in both Western (such as Denmark and Italy) and Eastern (Japan) cultures. This measure of general psychological adjustment was employed in 7 studies, most with children ages 11 and older. The other most commonly used self-report scale was the UCLA Posttraumatic Stress Disorder Reaction Index (UCLA PTSD-RI), which was used in 7 studies to screen for children’s and adolescents’ reactions to trauma. More specifically, the UCLA PTSD-RI is a self-report questionnaire that assesses the frequency of occurrence of PTSD symptoms over the past month and maps directly onto DSM intrusion, avoidance, and arousal criteria [57]. Measures that were employed for children ages 6 and older included the Center for Epidemiologic Studies Depression Scale (CES-D) and the Depression Self Rating Scale for Children (DSRS-C). These measures were used in 3 studies. For children ages 7 and older, the Child Depression Inventory (CDI) was used in 5 studies (one was the short form). For children ages 8 and older, scales included the Impact of Event Scale-Revised (IES-R), which was used in 5 studies and the Child PTSD Symptom Scale (CPSS) as well as the Child Revised Impact of Events Scale-13 (CRIES-13) used in 3 studies. Numerous other measures were used in only one study (e.g., the Derogatis Brief Symptom Inventory (BSI), the Multidimensional Anxiety Scale for Children Second Edition™ (MASC 2™), the Screen for Child Anxiety Related Disorders (SCARED), the Patient Health Questionnaire modified for adolescents (11–17) (PHQ-A), and the Short Cognitive Assessment System).

Of note is that certain commonly measured constructs such as depression and anxiety were measured using multiple different instruments (e.g., CDI, CES-D, PHQ-A, DSRS-C, and the Mood and Feelings Questionnaire to measure depression and the Anxiety Disorder Interview Schedule for Children, MASC-2, SCARED, and the Spence Children’s Anxiety Scale to assess anxiety). Self-efficacy was also measured in multiple studies using different instruments. Working definitions of constructs such as anxiety and depression varies from tool to tool. As a result, one should be cautious about making cross-comparisons of diagnostic terms and findings. Moreover, cultural/racial validity of assessment tools needs to be considered as the majority are established using population representation processes in which Whiteness is the norm. It is also important to note that these instruments are being used in various cultural environments such as collectivistic, communal/intergenerational, and individualistic. Particularly when studies are conducted with children in the same age range and culture, it is vital for researchers to select the same measures to more easily compare results. As the UCLA PTSD-RI and the SDQ were in the most studies, researchers are encouraged to consider these for future work in this area and to conduct additional norm-based studies.

### 3.5. Features of the Treatments

Approximately 31% of the studies evaluated the efficacy of CBT or its components (e.g., thought stopping; behavior activation), 16% evaluated TF-CBT or its treatment components (e.g., narrative exposure), and 16% of the studies used EMDR to treat associated posttraumatic stress symptoms and/or internalizing concerns associated with exposure. Additional interventions included Acceptance and Commitment Therapy (ACT), Catastrophic Stress Intervention (CSI), Cognitive Behavioral Intervention for Trauma in Schools (CBITS), Grief and Trauma Intervention (GTI) for Children, Narrative Exposure Therapy (NET), Psychological First Aid, Solution Focused Brief Therapy, various mindfulness strategies, and yoga and/or aerobic exercise education [10,11,20,26,28,34,47]. Finding common core elements across these therapies in order to evaluate their effectiveness to reduce traumatic stress in children and adolescents exposed to natural or biologic disaster is a crucial next step.

The interventions were primarily delivered in a group versus individual format, which makes practical sense when trying to reach as many effected children and adolescents as possible right after a disaster has occurred. TF-CBT was the most common intervention to be provided to individual children, such as in a treatment study conducted in Puerto Rico for trauma-exposed children after experiencing a hurricane or earthquake [22]. An example of a group format was in the CSI study following Hurricane Hugo in South Carolina [10]. Students met in a larger group of approximately 150 and broke out in smaller groups of 10–16 students each.

The dose of treatment varied greatly across studies. Specifically, the number of sessions ranged from one to 12 sessions, with many offering six. The length of sessions ranged from as short as 20 min to as long as 4 h, also the treatments commonly included sessions lasting for 45 min to 2 h. Studies varied greatly in intensity, i.e., how often the intervention material was presented, ranging from multiple times per week to every few months. One Chinese study provided treatment as often as 4 times per week for a month, with significant positive effects during the COVID-19 pandemic [28]. Comparison across studies would be quite valuable on this aspect of treatment delivery as it relates to cost effectiveness. The type of facilitator for treatments included psychologists, advanced practice psychiatric nurses, other mental health professionals as well as trainees.

### 3.6. Findings

Overall, there was a significant decrease in posttraumatic stress symptoms, distress, anxiety, and depression regardless of whether the patient received CBT, TF-CBT, or EMDR. Effect sizes were large in some of these studies, such as when providing individual therapy using TF-CBT. A large group after-school intervention also reported significant reductions in mental distress compared to students in the control condition. With regard to developmental considerations, at least one study found the opposite effect for EMDR among younger children with increased distress and anger, but other studies that included young children using TF-CBT or Psychological First Aid were determined to be therapeutic [16,22,46]. As there were so few studies with young children as participants, further research is needed to determine any differences in findings based upon developmental level.

There was not a significant decrease in anxiety and depression with yoga, cognitive fear-reduction, emotion-based drawing, and community health education. It seems as if a lack of selection of a standardized and well-validated intervention such as TF-CBT or even of less well-studied standardized treatments was associated with lower likelihood of significant effects.

Effect sizes were not reported for many studies, making it difficult to evaluate the impact of the intervention, i.e., small, medium, or large. An exception was for the Puerto Rican study of the individual intervention of TF-CBT for children exposed to a hurricane or earthquake; this study reported a large effect size for their sample of 56 [22]. There is also the issue of comparing disparate outcome constructs and measures so that conducting meaningful meta-analyses is not feasible with this collection of studies in the area thus far.

### 3.7. Follow-Up for Outcomes

These studies all contained immediate post-intervention assessments, but the number and timing of their follow-up varied. Follow-up data was provided for 44% of the studies included in this review. Follow-up data collection ranged from 1 month to 4 years with the modal duration being between 3 and 6 months. Of the 44%, 85% of the studies indicated that treatment gains were maintained. In the Hardin et al. study, treatment effects for CSI in response to a hurricane between the intervention and control groups dissipated after 24 months (i.e., were not found at 30 and 36 months) [10]. However, in the Giannopoulou et al. study evaluating the use of CBT in response to an earthquake decreases in posttraumatic stress symptoms were maintained at a 4-year follow up [37].

## 4. Discussion

Forty-five studies were identified that investigated the efficacy of intervention in response to natural and biologic disasters in children and adolescents. The most commonly implemented interventions were CBT, TF-CBT, and EMDR. Almost one-third of the studies evaluated the efficacy of CBT or one of its components (e.g., thought stopping; behavior activation). A sizable percentage (16%) evaluated TF-CBT or its treatment components (e.g., narrative exposure), and 16% used EMDR to treat associated posttraumatic stress symptoms and/or internalizing concerns associated with exposure. There were numerous other standardized interventions, including Acceptance and Commitment Therapy (ACT), Catastrophic Stress Intervention (CSI), Cognitive Behavioral Intervention for Trauma in Schools (CBITS), Grief and Trauma Intervention (GTI) for Children, Narrative Exposure Therapy (NET), Psychological First Aid, and Solution Focused Brief Therapy [10,11,20,26,28,34,47]. Twenty studies evaluated the efficacy of the intervention on posttraumatic stress symptoms with seven using the UCLA PTSD Index to measure posttraumatic stress symptoms. The UCLA PTSD Index and SDQ were the most commonly used measures. Overall, there was a significant decrease in posttraumatic stress symptoms, distress, anxiety, and depression regardless of whether the child or adolescent received CBT, TF-CBT, or EMDR. There was not a significant decrease in anxiety and depression with less standardized interventions such as yoga, cognitive fear-reduction, emotion-based drawing, and community health education.

### 4.1. Recommendations

Core principles of disaster interventions need to be identified, as there seems to be no single best practice approach. This will provide children and adolescents with a broader range of possible treatment options and providers. Considerations for choice of intervention are guided by many factors, including the specialty training of available clinicians, a focus on prevention vs. intervention, individual vs. group treatment, and accessibility. When referring professionals or the behavioral/mental health providers are seeking to choose an intervention in response to natural/biologic disasters, they could utilize this review’s table for comprehensive lists of intervention used by type and outcome. Additional investigation is needed for other types of therapies such as ACT and mindfulness strategies, etc., as there does not seem to be enough research that has been done to determine their effectiveness for the treatment of children who have post-traumatic stress symptoms following a natural disaster. In terms of study populations, future research should seek to include younger children (i.e., including preschool-aged children) as evidence exists that trauma-informed treatments such as TF-CBT can be effective for children as young as three years old who are able to remember details of the event. Also, studies should be conducted in continents other than North America, such as Africa, for which only one study was located for our review that provided treatment for children affected by AIDS. Ethnicity/race data should be collected for all studies and should not be collapsed across categories. Larger samples will provide the degrees of freedom to explore any racial/ethnic group differences in response to the elements of interventions to treat/prevent PTSD, i.e., to allow for moderation analyses. Finally, in order to reach a greater number of children and adolescents, future research should also consider the methodology of the larger-scale studies, such as group interventions and on-line therapy options.

It is recommended that future studies examine and dismantle specific treatment components that are shared across empirically-supported treatments to determine what is essential. Additional research is needed to determine the treatment modality (individual vs. group), intervention setting, length of therapy, and timing of intervention delivery that is most effective and compatible given the culture, resources, and type of disaster.

From a public health standpoint, effort should be focused on strategies that mitigate the impact of disasters on youth and families and on preparedness. This review underscores the importance of using the mitigation phase to identify the most cost-effective and culturally appropriate way to reach the greatest number of youth building on a community’s strengths and removing barriers. This review assists with everyday readiness as it provides a single source of interventions that has been used in the response and recovery phases and serves as a roadmap for needed considerations for methodological design. It is recommended that a repository of disaster-related resources exist for the behavioral health clinician to expedite post-disaster response. Such a repository will allow for the efficient design of developmentally and culturally appropriate interventions and measurement of treatment response with consideration of the disaster type.

### 4.2. Limitations

Despite some broad-sweeping generalizations drawn from this body of research, more fine-grained conclusions cannot yet be made due to the vast methodological differences, limited consensus on how constructs of interest should be measured, and lack of validated instruments in various populations and cultures. These studies demonstrated marked variability in passage of time between exposure and intervention. Some studies took a modular approach (e.g., thought stopping; behavior activation) while others evaluated the intervention effects as a whole. There were both individual and group interventions. The treatments were conducted in a variety of locations (e.g., school, on-line), and the provider type delivering the intervention was mixed. The authors encountered difficulty in conducting any meaningful meta-analyses due to lack of consistent measures used across studies. As many of the studies had small sample sizes, testing more complex models including mediation and moderation was not possible. Furthermore, self-selection bias must be considered when interpreting the findings of these studies. Those who participated in these studies might not represent the vast majority of those affected by natural disasters as these individuals were able to navigate barriers such as challenges with transportation, access to the internet, connection with mental health providers, and stigma that might be related to accessing mental health services. This might also have influenced the lack of inclusion of very young children and the infrequency of including younger children in general. Not all young children are in formal preschool programs in which trauma-informed interventions could be offered. The generalizability of this collection of studies is therefore limited by child age, geographic region, and by racial/ethnic groups with lack of representation of minorities across multiple countries. Due to the nature of the randomness of natural and biologic disasters, it is difficult to plan to replicate even the most promising methodologies. However, this is why preparedness is so important in effective behavioral health pediatric disaster response. Finally, a limitation of all systematic reviews is selection bias because of publication bias. Data from studies with statistically significant results are more likely to be published, limiting the scope of research in the area. This is particularly a problem in research with children and adolescents conducted after a natural disaster due to smaller samples reducing power to detect significance when effect sizes are not large.

### 4.3. Conclusions

This review provides compelling evidence for the use of CBT, TF-CBT, and EMDR for youth in response to natural/biologic disasters. Despite limitations of a research area that is broadly defined with inconsistent measurements and a range of treatment approaches, this review underscores the importance of devoting efforts and resources to mitigate the psychological impact of a natural disaster, engage in everyday readiness, and prepare for future catastrophic events. The review’s findings have notable implications not just for clinical practice, but also for policy development and future research studies. A take-home point is that evidence-based trauma-informed treatments for children and adolescents implemented via large group and internet-based interventions are viable for real-world applications with the broadest reach. While designing these response and recovery interventions, limitations of past research can be minimized by selecting empirically validated treatments, reaching out to the broadest possible age range and families from all affected racial/ethnic groups, selecting measures with established reliability/validity, and planning for follow-up assessments. Careful prior preparation will maximize the potential benefits of future trauma-informed interventions for children and adolescents affected by natural and biologic disasters.

## Figures and Tables

**Table 1:** Imported Abstracts by Database.

Database	Results
Cochrane Trials and Reviews	407
EBSCO CINAHL	480
Ovid Medline	715
Ovid PsycInfo	373
Hand Searching	4
Total	1979
Total with duplicates removed	1552

**Table 2:** Psychological Interventions Implemented in Response to Natural/Biologic Disasters.

Study	Disaster Type Location	Ages Studied (years) Sex (%, n=) Race/Ethnicity (%, n=)	Sample Size	Measures	Intervention(s)	Summary of Findings
N. Pityaratstian (2007) [9]	Natural disaster—Tsunami Thailand	Range: 9–15 59.4% female (n = 95) Not U.S. based.	N = 160	CRIES-13	**Quasi-experimental (no control)** 2 days, 2-h sessions that addressed avoidance and interacting with others facilitated by a mental health professional	No difference in CRIES scores before or after intervention (2 weeks after) unless separated into cohorts (listed below) Separated cohort into 2 groups for analysis, those with a CRIES score above and below cutoff (CRIES+ve/CRIES-ve) CRIES+ve group had significant decreases in Total, Intrusion, and Arousal scores CRIES-ve group had significant increases in Total, Intrusion, and Avoidance Scores Significant decrease in overall posttraumatic symptoms over the course of intervention once adjusted for participants in CRIES+ve (high pre-intervention CRIES score group)
S. Hardin (2002) [10]	Natural disaster—Hurricane Hugo South Carolina, United States	Range: 13–18 50% female (n = 515) 44% white (n = 453) 54% black (n = 556)	N = 1030	Derogatis Brief Symptom Inventory (BSI) Self-Efficacy Scale Social Support Scale Negative Life Events Checklist	**Randomized Control Trial** Catastrophic Stress Intervention (CSI) designed for increasing self-efficacy and social support Intervention was for 3.5 to 4 h in a large group followed by 60 min at a time in groups of 10–16 adolescents three times a year for three years. Control did not receive an intervention.	Individuals in CSI intervention had less mental distress than control Significant intervention effect when controlling for age, sex, etc. at each time point. Effects of CSI intervention effectiveness dissipates over time, found at the 24 month period; the longer the gap between trauma and intervention, the less of the effect From 12 months to 24 months (during the intervention), CSI participants mental distress decreased less than assigned/nonparticipants or control group
C. Catani (2009) [11]	Natural Disaster—Tsunami North-East Sri Lanka	Range = 8–14 45% female (n = 14) Not U.S. based.	N = 31	UCLA PTSD Index for DSM-IV	**Quasi-experimental (no control)** Narrative Exposure Therapy (NET), 6 sessions of 60 to 90 min within 2-week period MED-RELAX (meditation—relaxation), 6 sessions of 60 to 90 min within 2-week period	In both groups, PTSD symptoms significantly reduced after 1 month post-test and remained stable over time (6-month follow-up) 81% recovery (not meeting UCLA PTSD index threshold for PTSD) in KIDNET group and 71% for MED-RELAX group 6 months post-intervention Significant reduction in impairments in psychosocial functioning in both groups after 6 months
M. Adúriz (2009) [12]	Natural Disaster—Flood Argentina	Range: 7–17 51% female (n = 63) Not U.S. based.	N = 124	SUD CRTES	**Quasi-experimental (no control)** EMDR-IGTP (Integrative Group Treatment Protocol) for 2 sessions each for 2 h with groups of 18 students 3 month follow-up with another 2 h EMDR-IGTP session	EMDR showed significant decrease in SUD scores from pre to post and in CRTES scores which were maintained at 3-month follow-up Girls had higher scores than boys at pre-intervention, decreased at post-intervention, with a greater number of girls in the high distress category
A. Bazzano (2022) [13]	Pandemic—COVID-19 New Orleans, LA	Range: 11–14 46% female (n = 40) 55 (67.1%) African American	N = 86	PHQ-A SCARED	**Randomized Control Trial** Intervention was yoga education and mindfulness for 8 weeks once a week for 45 min Waitlist control consisted of their course schedule as usual	No significant decrease in anxiety and depression in the intervention Control group had an increase in depressive symptoms but a decrease in anxiety Significant difference in PHQ-A but not SCARED (lower PHQ-A values in yoga group than control) Time effect showed significant decrease in anxiety symptoms Possible effectiveness of yoga to manage symptoms
		19 (23.2%) white 6 (7.3%) Asian 2 (2.4%) multi-racial				
K. Kishida (2022) [14]	Pandemic—COVID-19 Japan	Range: 12–13 62% female (n = 74) Not U.S. based.	N = 120	SDQ GSESC-R Short CAS DSRS-C ASCA	**Quasi-experimental (no control)** Up2-D2 intervention (12 cognitive-behavioral and positive psychology sessions for 45–50 min each) designed as a prevention program targeting internalizing and externalizing problems	Improved anxiety, but not depression or anger At 2 month follow-up, anxiety was shown to be significantly decreased At 6 month follow-up, anxiety, general difficulties, emotional symptoms, and anxiety were significantly decreased General difficulties domain was also significantly decreased at post-assessment More improvement for those with higher baseline anxiety, especially at post-assessment
J. Schleider (2022) [15]	Pandemic—COVID-19 United States	Range: 13–16 88% female (n = 2158)	N = 2452	Beck Hopelessness Scale—Four-Item Version State Hope Scale—Agency Subscale CDI-SF COVID-19 Trauma Symptoms GAD-7 Dietary Restriction Screener	**Randomized Control Trial** Online SSI (single session intervention) for children with depression that teaches behavioral activation, 2 half hour sessions that was either Project ABC/BA-SSI or Project Personality/GM- SSI Project ABC/BA-SI (behavioral action) promotes values-based activity engagement to elicit pleasure and accomplishment Project Personality/GM-SSI (growth mindset) an online program teaching that personal traits are malleable, which has prevented and reduced adolescent depression Placebo SSI without the promotion of either of these values—not an active intervention	Relative to the control, both SSIs resulted in increased perceived agency and decreased hopelessness, decreased depressive symptoms, and restrictive eating behaviors but did not differ between each other significantly at 3 month follow-up Participants in GM-SSI had significant decreases in anxiety, but not BA-SSI BA-SSI increased perceived agency more than GM-SSI at 3 month follow-up GM-SSI had a larger decrease in anxiety symptoms than the BA-SSI at 3 month follow-up Active interventions are capable of moderately reducing depressive symptoms, restrictive eating, and anxiety and increasing perceived agency
C. Trentini (2018) [16]	Natural Disaster—Earthquake Umbria, Italy	Range: 5–13 % Sex not given Not U.S. based.	N = 332	Emotional Thermometers 5 CRIES-13	**Quasi-experimental (no control)** EMDR- Integrated Group Treatment Protocol (IGTP) with 60 to 90 min sessions for a total of 8 sessions designed to process traumatic experiences of those with acute traumatic stress	Older children had reduction in distress and anger Younger children had increased distress and anger Females had a greater reduction of distress, anxiety, and need for help Greater improvement in depressive symptoms in males than females Authors concluded that the intervention was effective
Y. Hardayati (2019) [17]	Natural Disaster—Earthquake Indonesia	Range: 15–17 80% female (n = 90) Not U.S. based.	N = 112	Pasaribu Derived Questionnaire	**Randomized Control Trial** Intervention consisted of thought stopping therapy and a nursing intervention (4 meetings in total) Control was given the nursing intervention only (2 meetings in total)	Significant gender effects in the ability to regulate negative thoughts that played a role in the effectiveness of the intervention Intervention found to better enable participants to control negative emotions than control
K. Stasiak (2016) [18]	Natural Disaster—Earthquake New Zealand	Range: 7–15 53% female (n = 22) Not U.S. based.	N = 42	DSM-IV criteria ADIS-C/P SCAS MFQ-S Short Version CHU9D CGAS	**Quasi-experimental (no control)** BRAVE-ONLINE (CBT) consisting of 10 sessions lasting between 20–45 min	Significant reductions in anxiety and depressive symptoms in severity and number of participants who had clinically significant anxiety Most participants no longer met the DSM-IV criteria for anxiety at post-intervention Those who still had a diagnosis of anxiety had a mean decrease in severity All participants no longer met the DSM-IV criteria for PTSD by end of intervention and maintained at 6 month follow-up.
O. Karairmak (2008) [19]	Natural Disaster—Earthquake Turkey	Range: 4th through 8th grade 32% female (n = 85) Not U.S. based.	N = 266	FSSC	**Randomized Control Trial** Activity based cognitive fear reduction 3 times for 3 weeks each 90-min groups Control group had sessions with structured activities and games 3 times for 3 weeks each 90-min groups	Girls were/are more fearful than boys and significant gender differences were revealed Girls also had higher scores on all subscales of the FSSC compared to boys Cognitive fear-reduction was not effective to lessen fears No interaction effect between gender x group
L. Jaycox (2010) [20]	Natural Disaster—Hurricane Louisiana	Range: 11.6 ± 1.4 56% female (n = 66) Assigned by School for Treatment School 1: 74% African American School 2: 90% Caucasian	N = 118	Disaster Experiences Questionnaire UCLA PTSD Reaction Index for DSM-IV CDI SSSC SDQ	**Randomized Control Trial** TF-CBT (12 sessions) CBITS (11–13 sessions)	PTSD improved faster and more dramatically for CBITS group than the TF-CBT group Significant decrease in depressive symptoms in CBITS group CBITS is more accessible to families More children at risk for PTSD in TF-CBT group than CBITS Both significantly decreased PTSD symptoms even after 10 month follow-up
		School 3: 95% African American				
I. Nicolaidou (2021) [21]	Pandemic—COVID-19 Cyprus	Range: 9–10 49% female (n = 20) Not U.S. based.	N = 41	SCAS SUS	**Quasi-experimental (no control)** Web-based delivery of formal instruction on management of emotions, stress, and resilience consisting of 6, 80-min sessions Control did not receive the intervention	Slight but non-significant reduction in symptoms associated with OCD after intervention Slight but non-significant reduction in anxiety disorders Improved children’s ability to recognize emotions and engage in stress management.
R. Orengo- Aguayo (2022) [22]	Natural Disaster –Hurricane & Earthquake Puerto Rico	Range: 5–18 48% female (n = 47) Did not have Race/Ethnicity data.	N = 56	CPSS-5 RCADS-SV	**Quasi-experimental (no control)** TF-CBT was delivered for once a week for 8 weeks each 50–60 min individual sessions	Effective at reducing PTSD, depression, and anxiety symptoms in trauma-exposed youth
C. Malbouef-Hurtubise (2021) [23]	Pandemic—COVID-19 Canada	Mean: 11.3 50% female (n = 11) Not U.S. based.	N = 22	Mindful Attention Awareness Scale (BASC III)	**Quasi-experimental (no control)** Randomized to 2 interventions Online emotion-based directed drawing intervention for 5 weeks for 45 min each week Online mandala drawing intervention for 5 weeks for 45 min each week	While means differed, not significant between groups for inattentiveness or inattention, anxiety, depression, hyperactivity, or mindfulness Significant decrease in hyperactivity for whole sample
C. Malbouef-Hurtubise (2021) [24]	Pandemic—COVID-19 Canada	Mean: 8.18 43% female (n = 20) Not U.S. based.	N = 37	BASC	**Quasi-experimental (no control)** Randomized to either 5-week intervention P4C (philosophy for children) intervention with weekly sessions for 1-h each where COVID-19 themes were discussed to foster basic psychological need satisfaction, and more specifically the need for autonomy MBI (mindfulness based intervention) with weekly sessions for one hour each to discuss mindfulness and meditative practices	Both interventions improved the mental health of elementary school students MBI improved introspection and improved basic psychological needs more than the P4C intervention P4C improved mental health through reflection and improved anxiety and inattention P4C intervention may be more impactful to reduce inattentive symptoms whereas MBI may be more useful to improve basic psychological needs in the current context of the COVID-19 pandemic
T. Tang (2015) [25]	Natural Disaster—Typhoon Taiwan	Range: 12–15 54% female (n = 45) Not U.S. based.	N = 83	C-IES-R MASC-T CES-D	**Randomized Control Trial** EMDR group was facilitated for 3 months, 3 months post- typhoon. They also received a psychoeducational component identical to the control group weekly for a total of 4 sessions TAU group consisted of weekly 40 min psychoeducational sessions on PTSD.	EMDR more effective than TAU for reducing severity of disaster-related anxiety EMDR alleviated general anxiety symptoms Self-reported depression improved through the EMDR intervention
W. Xu (2021) [26]	Pandemic—COVID-19 Fuijan Province, China	Range: 12–19 % female unknown Not U.S. based.	N = 90	GHQ-12 WEMWBS AAQ-II	**Randomized Control Trial** 8-week ACT (acceptance and commitment therapy) intervention and aerobic exercise provided 3 times a week for 40–60 min per session Control group didn’t receive any intervention	Increase in well-being and psychological flexibility, decrease in psychological distress, and increase in psychological flexibility in intervention group compared to control Aerobic intervention was found to improve the positive psychological state while the ACT intervention decreased the negative psychological state Follow up was conducted after one post-intervention month
E. Lazzaroni (2021) [27]	Pandemic—COVID-19 Italy	Range: 13–24 84% female (n = 42) Not U.S. based.	N = 50	IES-R STAI-Y Emotion Thermometer PTGI	**Quasi-experimental (no control)** 3 group meetings of 1 h each following the brief EMDR protocol created by the Critical Incident Stress Management and EMDR protocols for Acute and Recent Traumatic Events with the butterfly hug	Highest reduction in intrusiveness and hyperarousal domains EMDR reduced the emotional impact of the stressful event Perceived positive change globally in the EMDR intervention Clinical improvement due to early treatment with EMDR
J. Li (2021) [28]	Pandemic—COVID-19 Anhui Province, China	Mean: Intervention: 15.18 ± 1.37 48% female (n = 29) Control: 15.24 ± 1.38 47% female (n = 27) Not U.S. based.	N = 128	SAS Positive and Negative Affect Scale	**Randomized Control Trial** Interventions were one month, 4 times each week, every 2 days for both Intervention was solution-focused brief therapy-based group intervention with a short video health education related to the pandemic Control group was health education in the community lecture due to pandemic	The intervention group had significantly decreased SAS scores with a greater effect in the intervention group than the control Intervention had higher positive affect score and lower negative affect than the control group post-intervention. Authors concluded that the intervention was effective
M. Shooshtary (2008) [29]	Natural Disaster—Earthquake Bam, Iran	Range: 11–20 Control: 60% female (n = 20) Intervention: 53% female (n = 72) Not U.S. based.	N = 168	IES-R GHQ-28	**Randomized Control Trial** Intervention was a CBT session that lasted for 2 h each over the course of 4 weeks for a total of 4 sessions Comparison group received no treatment during the study	Significant reduction in posttraumatic stress symptoms in intervention in all 3 PTSD symptom categories (intrusion, avoidance, and arousal) The CBT group evidenced significant improvement in anxiety, depression, and dissociation at the 6 month follow up, and in PTSD and dissociation at the 12 month follow up
J. Zhang (2021) [30]	Pandemic—COVID-19 Shandong Province, China	Range: 12–18 47% female (n = 75) Not U.S. based.	N = 160	Healthy Kids Resilience Assessment SDS SAS PSQI	**Randomized Control Trial** Intervention group received routine community health education and counseling for one hour each week, once a week for 8 weeks Control group received routine community health education	No difference observed between the intervention and control group; from pre to post, there was a significant decrease in anxiety and depression scores No difference in sleep scores or psychological resilience scores after 2 month follow up
C. Chemtob (2002) [31]	Natural Disaster—Hurricane Hawaii, USA	Range: 6–12 61% female (n = 151) “No significant difference” for ethnicity effects. Race/Ethnicity not provided.	N = 248	Kauai Recovery Inventory (KRI) Child PTSD Reaction Index (CRI)	**Randomized Control Trial** 1 of 3 sequential groups who received manual-guided 4 weekly sessions with a therapist In each cohort, 1 child out of 4 was assigned to individual therapy and the rest to group therapy	Group and individual treatment reduced trauma symptoms and facilitates recovery ‘equally’. No difference between group and individual treatment across cohorts Fewer children dropped out of group treatment than individual treatment
J. Chen (2021) [32]	Pandemic—COVID-19 Zheijang Province, China	Range: 13–16 49% female (n = 35) Not U.S. based.	N = 72	SAS PANAS PWBS	**Randomized Control Trial** The experimental group participated in an aerobics exercise course and mindfulness meditation training, once a day for 5 weeks for 30 min each with health education support Control group received health education support	Significant difference in SAS score between experimental and control; experimental had a greater significant score decrease than control The intervention group had a stronger decrease in negative scores (anxiety, negative emotion) and stronger increase in positive scores (well-being, positive emotion) across all measurements compared to control Overall well-being increased in both groups, but more so in the intervention (statistically significant) Authors’ concluded that the intervention was effective
J. Cohen (2021) [33]	Pandemic—COVID-19 Puerto Rico	Range: “Children” % female unknown Not U.S. based.	N = 222	CPSS	**Randomized Control Trial** Randomized to either TF-CBT or usual care	Significantly greater improvement in PTSD than usual care and maintained after long- term follow up (1 year)
W. Duan (2022) [34]	Pandemic—COVID-19 Wuhan City, China	Range: 10–12 57% female (n = 43) Not U.S. based.	N = 76	GAD-2 Mini-Q-LES-Q	**Quasi-experimental (no control)** All received ACT intervention for 10 weeks	Adolescents who received crisis events and used the intervention, had an improvement in QOL and reduction in anxiety symptoms after 3 months
I. Fernandez (2007) [35]	Natural Disaster—Earthquake Molise, Italy	Range: 7–11 Sex: not provided Not U.S. based.	N = 22	SCID-1	**Quasi-experimental (no control)** All received EMDR therapy for 30 to 90 min with 3 cycles of treatment with variable amounts of EMDR for each child	Significant reduction in PTSD symptoms within 1 year of EMDR Decrease in avoidance, intrusiveness, and arousal and proportion of sample meeting PTSD criteria
S. Gadari (2022) [36]	Pandemic—COVID-19 Southeastern Iran	Range: 9–10 100% female (n = 80) Not U.S. based.	N = 80	Children’s Social Self-Efficacy in Peer Interaction Scale	**Randomized Control Trial** Intervention was an online intervention where there were video workshops and lessons about self-efficacy with 12 sessions for 6 weeks, twice a week Control group did not receive the intervention, but did after the completion of the study	Resilience training was effective in promoting social efficacy of female children Higher self-efficacy in experimental than control Intervention given during COVID
I. Giannopoulou (2006) [37]	Natural Disaster—Earthquake Greece	Range: 8–12 55% female (n = 11) Not U.S. based.	N = 20	CRIES DSRS SDQ	**Randomized Control Trial** 6 week group CBT for a total of 6 weekly 2-h sessions Group 1 was the immediate treatment group who received treatment 2 months post-earthquake Group 2 was a delayed treatment group who received treatment 4 months post-earthquake	Short-term group CBT is effective in reducing overall PTSD symptoms across all PTSD symptom clusters and depressive symptoms with mean scores of PTSD significantly reduced at an 18-month follow up and maintained at 4 year follow up
G. Maslovaric (2017) [38]	Natural Disaster—Earthquake Valle del Tronto, Italy	Range: 13–20 44% female (n = 51) Not U.S. based.	N = 116	IES-R	**Quasi-experimental (no control)** 3 EMDR sessions for adolescents	EMDR was able to reduce the negative emotional load associated with the event EMDR also reduced the PTSD symptoms present Reduction in hyperarousal scale at 3-month follow up
M. Karadag (2021) [39]	Pandemic—COVID-19 Turkey	Range: 9.07 ± 0.8 55% female (n = 98) Not U.S. based.	N = 178	STAIC CPTS-RI	**Randomized Control Trial** The intervention group carried out the activities (EMDR and CBT) in the treatment guide three times in total, once every 2 days with each session taking an average of 20 min Control group did not receive an intervention	EMDR self-help intervention group had significant decrease on trauma measure scores at 1 month follow up Control had increase in the trauma measures scores
T. Kubo (2022) [40]	Pandemic—COVID-19 Japan	Range: 12–15 47% female (n = 117) Not U.S. based.	N = 248	DSRS-C FCV-19 STAIC	**Randomized Control Trial** Groups divided by each grade; first grade: control group, second grade: announcement group, third group: intervention group Intervention group had 50 min sessions, with 15 min self- monitoring and 35 min psychoeducation Announcement group had an announcement about how pandemic- related stress effects mental health for 15 min. Control group had no such intervention/announcement.	Depression significantly reduced in intervention group No significant difference in fear of COVID-19 or trait anxiety
M. Keypour (2011) [41]	Pandemic—AIDS Iran	Range: 13–18 40 % female (n = 14) Not U.S. based.	N = =34	GHQ-28 SDQ Family Assessment Device (FAD)	**Quasi-experimental (no control)** Individual CBT intervention for 8 weekly sessions each lasting 90 min	Intervention was effective and sustained results at 3-month follow up on GHQ and SDQ No effect on social behavior Peer group problems deceased
Mueller (2010) [42]	Pandemic—AIDS Knysna, South Africa	Range: 8–18 48.1% female (n = 143) Not U.S. based.	N = 297	RSES SEQ-C CDI SDQ	**Randomized Control Trial** Make a Difference (MAD) About Art community-based intervention; 50+ sessions over the course of 6 months (art and education activities to build a sense of self-worth, self-concept, empowerment and emotional control) Control group (no MAD programming)	Attending the intervention was predictive of significantly higher self-efficacy but was not associated with differences in self-esteem, depression, or emotional/behavioral problems Double parental death exerted a powerful effect on child psychosocial health, eliminating the association between intervention attendance and higher self-efficacy An interaction was found between bereavement status and intervention attendance on child self-efficacy
Pityaratstian (2015) [9]	Natural disaster—tsunami Thailand	Range: 10–15 72.2% female (n = 26) Not U.S. based.	N = 36	CRIES (Thai version) UCLA PTSD-RI CGI GAF	**Randomized Control Trial** 3-day, 2-h-daily CBT group format followed by 1-month of post-treatment with self-monitoring and daily homework Wait list control	No significant improvement in the CBT group at post-treatment Children who received CBT demonstrated greater improvement in symptoms of PTSD at 1-month follow up vs. the wait list group
Chen (2014) [43]	Natural disaster—earthquake Sichuan, China	Range: 14.50 ± 0.71 (mean ± SD) 68% female (n = 22) Not U.S. based.	N = 32	CD-RISC CRIES-13 CES-D	**Randomized Control Trial** 6-week, 1-h sessions, group-based CBT with the opportunity to share experiences, develop a narrative, and learn skills to cope with PTSD and depression 6-week, individual general supportive intervention counseled by volunteers trained in listening, reflection, and empathy; support and assistance was provided to adolescents with problems at home and school Non-treatment control group	CBT was effective in reducing PTSD and depressive symptoms and improving psychological resilience which was maintained at 3 month follow-up General support was more effective than no intervention in improving psychological resilience
Ruggiero (2015) [44]	Natural disaster—tornado Joplin, MO and Alabama	Range: 14.50 ± 1.7 (mean ± SD) 53% female (n = 523) 62.5% white (n = 617) 22.6% black (n = 223) 3.8% other (n = 38) 2.7% Hispanic (n = 27)	N = 987	NSA PTSD module NSA Depression module	**Randomized Control Trial** Bounce Back Now (BBN) modular, web-based psychoeducation intervention for disaster-affected adolescents and their parents addressing post-disaster mental health BBN plus an additional 7-module adult self-help intervention targeting parents’ mental health and substance use problems Assessment only web-based control condition	Significantly fewer PTSD and depressive symptoms for adolescents in the experimental vs. control condition at 12-month follow-up, controlling for baseline levels Time x condition interaction indicated that BBN was favored over BBN + parent self- help condition for PTSD symptoms but not depressive symptoms
Martin (2015) [45]	Natural disaster—Earthquake Lorca, Spain	Range: 9–14; % female not given Not U.S. based.	N = 89	SCARED STAI for children CDI Resilience Scale	**Randomized Control Trial** Assigned to intervention group based on diagnosis and severity of symptoms Normalization intervention consisted of helping patients normalize their situations and view symptoms as a normal reaction to a traumatic event that are most likely to disappear with time Brief group trauma-focused psychotherapy treatment (for those with mild to moderate elevation of symptoms) received 5 weekly sessions Individual trauma-focused psychotherapy treatment (for those diagnosed as having PTSD or severe adjustment disorders) received 10 weekly sessions Referral to mental health center for prior disorder or new severe disorder post-earthquake	Rates of symptom resolution and improvements on all scales (PTSD, depression, anxiety and resilience) demonstrated clinically and statistically significant improvement in all treatment groups, which were maintained at 1 month follow-up Normalization can adequately treat mild symptoms of depressive and anxiety disorders after natural disasters such as earthquakes
Cain (2010) [46]	Natural disaster—Hurricane New Orleans, LA	Range: 5–15 53% female (n = 52) 95% African American (n = 94) 5% biracial (n = 5)	N = 99	CPTS-RI	**Quasi-experimental (no control)** Assigned to 6-week Psychological First Aid (PFA) group intervention	Post-intervention scores reflected a statistically significant improvement in PTSD symptoms among the sample Significant improvements post-intervention on levels of fear, feeling less alone and more understood, less startle responses, fewer memory problems, and decreased likelihood to feel upset when reminded of the hurricane
Salloum (2012) [47]	Natural disaster—Hurricane New Orleans, LA	Range: 6–12 44.3% female (n = 31) 100% African American (n = 70)	N = 70	MFQ UCLA PTSD-RI Extended Grief Inventory 5-response Likert format MSPSS CBCL	**Randomized Control Trial** 10-week group of Grief and Trauma Intervention (coping skills and trauma narrative modules) with 1 individual session and 1 parent session 10-week group of Grief and Trauma Intervention (coping skills only) with 1 individual session and 1 parent session	Children in both groups demonstrated significant improvements in distress-related symptoms and social support With the exception of externalizing symptoms for GTI-C, improvements in distress related symptoms and social support were maintained up to 12 months post intervention Higher percentages of children migrated out of clinical symptom ranges and demonstrated more reliable improvement, and less reliable deterioration in the GTI- CN group versus the GTI-C group
Taylor (2011) [48]	Natural disaster—Hurricane New Orleans, LA	Range: 8–13 67% female (n = 4) 100% African American (n = 6)	N = 6	UCLA PTSD-RI ADIS-IV CASI ACQ-C CNCEQ DISC-PS	**Quasi-experimental (no control)** Assigned to 10-session StArT manual-based (CBT group) intervention designed for traumatized youth	All six participants no longer met criteria for PTSD at post-treatment assessment Results provide initial evidence for the efficacy of the StArT manual and suggest the feasibility of conducting the StArT manual in a school setting
Osofsky (2018) [49]	Natural disaster—Hurricane New Orleans, LA	Range: 15–17 56% female (n = 119) 81% Caucasian (n = 172 8% African American (n = 17) 5% Hispanic (n = 11) 4% Other (n = 8)	N = 212	Status Questionnaire for Students UCLA PTSD-RI	**Self-Selected Participation** Youth Leadership Program (YLP), which is a year long group community service project and mental health intervention (stress reduction and self-awareness activities) No YLP participation	Students who participated in the YLP, compared with peers who did not participate scored significantly higher on self-efficacy An interaction effect revealed that gains in self-efficacy also resulted in reduced trauma symptoms for both groups of students
Powell (2016) [50]	Natural disaster—Tornado Tuscaloosa, Alabama	Range: 8–12 52.9% female (n = 54) 80.2% African American (n = 82) 5.9% White (n = 6) 5.9% Latino (n = 6) 2.0% Native American (n = 2)	N = 102	YCI SDQ Community that Cares survey	**Randomized Control Trial** Classrooms were assigned to conditions based on the teacher’s timing of intervention to minimize academic disruption JoH (Journey of Hope) school-based intervention focused on group work, building coping skills, and enhancing protective factors Waitlist control group	Outcomes indicate that after participation in the JoH program, youth had increased coping skills and prosocial behaviors Findings provide preliminary evidence of the effectiveness of this broad-based post disaster intervention
Westerman (2017) [51]	Natural disaster—Flood Queensland, Australia	Range: 8–17 54% female Not U.S. based.	N = 26	ADIS-IV	**Quasi-experimental (no control)** Assigned to up to 10 sessions with the first 2 sessions consisting of psychoeducation about PTSD and therapy for parents and children; sessions 2–10 were TF-CBT trauma narrative sharing for children	Distress experienced by the children and adolescents decreased over the sessions as measured by the coding system that evaluated coherence (structure, orientation of events, normative elements), level of elaboration of the story (description, emotion, sense of self), and the evaluation of the story (causality, insight, and integration) Analysis using the coding system identified that the internal logic of the stories were maintained as the detail diminished, and the level of evaluation of causality, self- reflectivity, and integration into their life increased
Ito (2016) [52]	Natural disaster—Earthquake Tohoku region, Japan	Range: 15.36 ± 0.49 68% female Not U.S. based.	N = 22	IES-R CES-D	**Quasi-experimental (no control)** Assigned based on IES-R score greater than a cutoff point with intervention being one, 90-min CBT group intervention.	Significant improvement in all posttraumatic symptoms at post-intervention with the effects maintained at 4-month follow up No improvement in depressive symptoms was shown

Abbreviations = AAQ-II, Acceptance and Action Questionnaire–II; ACQ-C, Anxiety Control Questionnaire for Children; ADIS-IV, Anxiety Disorders Interview Schedule for DSM-IV; AESC, Anger Expression Scale for Children; BDS, Birleson Depression Scale; BERS-2, Behavioral and Emotional Rating Scale-2; BYI, Beck Youth Inventory; CASI, Childhood Anxiety Sensitivity Index; CASSS, Child and Adolescent Social Support Scale; CBCL, Child Behavior Check List; CDI, Children’s Depression Inventory; CD-RISC, Connor-Davidson Resilience Scale; CES-D, Center for Epidemiologic Studies Depression Scale; CGI, Clinical Global Impression Scale; CNCEQ, Children’s Negative Cognitive Error Questionnaire; CPSS, Child PTSD Symptom Scale; CPTS-RI, Child Post-Traumatic Stress Reaction Index; CRIES-13, Child Revised Impact of Events Scale-13; CROPS, Child Report of Post-traumatic Symptoms; CRTES, Child’s Reaction to Traumatic Events Scale; DISC-PS, Diagnostic Interview Schedule for Children-Predictive Scales; DTS, Davidson Trauma Scale; DSRS, Depression Self-Rating Scale; GAD-2, Generalized Anxiety Disorder Scale; GAF, Global Assessment of Functioning Scale; GHQ-28, Generalized Health Questionnaire-28; IES-R, Impact of Event Scale-Revised; MASC, Multidimensional Anxiety Scale for Children; Mini-Q-LES-Q, Mini Quality of Life Enjoyment and Satisfaction Questionnaire; MFQ, Mood and Feelings Questionnaire; MSPSS, Multidimensional Scale of Perceived Social Support; NSA, National Survey of Adolescents; PANAS, Positive and Negative Affect Scale; PROPS, Parent Report of Post-traumatic Symptoms; PSQI, Pittsburgh Sleep Quality Index; PSR, Provisions of Social Relations; PTGI, Post Traumatic Growth Inventory; PTSD-RI, Post-traumatic Stress Disorder Reaction Index; PWBS, Psychological Well-Being Scale; RSES, Rosenberg Self-Esteem Scale; SAS, Self-Rating Anxiety Scale; SCID, Structured Clinical Interview for DSM-IV; SCRED, Screen for Child Anxiety Related Emotional Disorders; SDQ, Strengths and Difficulties Questionnaire; SDS, Self-rating Depression Scale; SEQ-C, Self-Efficacy Questionnaire for Children; STAI, State-Trait Anxiety Inventory; STAIC, State-Trait Anxiety Inventory for Children; SUDS, Subjective Units of Disturbance Scale; TGIC, Traumatic Grief Inventory for Children; WEMWBS, The Warwick-Edinburgh Mental Wellbeing Scales; YCI, Youth Coping Index.

## Data Availability

No new data was obtained.
